# Validation of the cutoff values for the number of metastatic lymph nodes for esophageal cancer staging: a multi-institutional analysis of 655 patients in Japan

**DOI:** 10.1007/s10388-024-01084-6

**Published:** 2024-08-24

**Authors:** Koji Tanaka, Takeo Fujita, Yasuaki Nakajima, Akihiko Okamura, Kenro Kawada, Masayuki Watanabe, Yuichiro Doki

**Affiliations:** 1 Department of Gastroenterological Surgery, Graduate School of Medicine, Osaka University, Osaka, Japan; 2 Department of Esophageal Surgery, National Cancer Center Hospital East, Kashiwa, 6-5-1 Kashiwanoha, Kashiwa-Shi, Chiba 277-8577 Japan; 3 Department of Surgery, Edogawa Hospital, 2-24-18 Higashi-Koiwa, Edogawa-ku, Tokyo, 133-0052 Japan; 4 Department of Gastroenterological Surgery, The Cancer Institute Hospital of Japanese Foundation for Cancer Research, 3-8-31 Ariake, Koto-ku, Tokyo, 135-8550 Japan; 5 Department of Gastrointestinal Surgery, Tokyo Medical and Dental University, Tokyo, Japan

**Keywords:** Lymph node metastasis, Esophageal cancer, Prognosis, Staging

## Abstract

**Background:**

The number of metastatic lymph nodes (LNs) is an important prognostic factor for esophageal cancer, and N staging is important for prognostic stratification. The optimal cutoff values for clinical (cN) and pathologic N (pN) staging should be reconsidered following advances in neoadjuvant therapy.

**Methods:**

The study included 655 patients who underwent esophagectomy between January 2014 and December 2016 in four high-volume centers in Japan. Optimal cutoff values for the number of metastatic LNs in cN and pN staging were examined using X-tile, and their prognostic performance was validated using the Kaplan–Meier method.

**Results:**

The cutoff values were 1, 2, and 3 for cN staging and 1, 3, and 7 for pN staging. Prognosis was significantly better in patients with cN0 than in those with modified (m)-cN1 (*p* = 0.0211). However, prognosis was not significantly different among the patients with m-cN1, m-cN2, and m-cN3 disease. Prognosis was significantly different among the patients with pN0, pN1, pN2, and pN3 disease (pN0 vs pN1, *p* < 0.0001; pN1 vs pN2, *p* < 0.0001; pN2 vs pN3, *p* < 0.0001). In patients who received preoperative neoadjuvant therapy, prognosis, which was not significantly different among the patients with cN0, m-cN1, m-cN2, and m-cN3 disease (cN0 vs m-cN1, *p* = 0.5675; m-cN1 vs m-cN2, *p* = 0.4425; m-cN2 vs m-cN3, *p* = 0.7111), was significantly different among the patients with pN0, pN1, pN2, and pN3 disease (pN0 vs pN1, *p* = 0.0025; pN1 vs pN2, *p* = 0.0046; pN2 vs pN3, *p* = 0.0104).

**Conclusions:**

cN has no prognostic impact in patients who underwent preoperative treatment followed by esophagectomy, despite the optimization of cN classification. The conventional TNM8th pN classification is useful for predicting prognosis even for patients who have undergone preoperative treatment. The conventional cutoffs for metastatic LNs in the International Union against Cancer tumor node metastasis staging system are valid and can be effectively used in clinical practice.

## Introduction

Globally, esophageal cancer is the fifth and eighth most common cause of cancer-related deaths in men and women, respectively [1]. Despite the development of new treatment strategies and improvements in surgical approaches for esophageal cancer [2, 3], the 5 year overall survival rate is 15–35% and the prognosis remains dismal [4, 5].

Cancer staging systems that accurately predict prognosis in patients with esophageal carcinoma is critical for the selection of appropriate treatment strategies. The tumor node metastasis (TNM) cancer staging system developed by the American Joint Committee on Cancer is widely used for the prognostic stratification of patients with esophageal cancer. The N classification is particularly important for a variety of carcinomas [6], and numerous studies have demonstrated the number of lymph node (LN) metastasis as one of the most important prognostic factors for esophageal cancer [7–9].

Despite the use of pretreatment imaging modalities such as positron emission tomography (PET)–computed tomography (CT) and endoscopic ultrasonography and the improved quality of CT in recent years, the utility of imaging modalities for the diagnosis of LN metastasis remains insufficient [10, 11]. Although clinical and pathologic N stages (cN and pN, respectively) have been separately classified since the 8th edition, the cutoff number of LNs is the same for both cN and pN staging in the International Union against Cancer (UICC) TNM classification. However, following recent advances in neoadjuvant therapy, it remains unclear if the same cutoff should be used for the number of pathologic LNs and the number of pretreatment LNs in determining the N stage.

In this retrospective study, we aimed to confirm the cutoff value for the number of LN metastasis for the N staging system in a large cohort of patients with esophageal cancer who underwent esophagectomy.

## Patients and methods

### Patients

Data were collected from the medical records stored in an esophageal cancer database, which included patients who underwent surgery in four high-volume centers in Japan. In the present study, 962 consecutive patients who underwent surgery between January 2014 and December 2016 and met the following criteria were included: (1) subtotal esophagectomy and mediastinal lymph node dissection were performed, (2) successful curative resection (R0), (3) primary tumor located mainly in the thoracic esophagus and (4) cM0 and pM0.

Preoperative chemotherapy or preoperative chemoradiotherapy was administered according to each institution’s policy, taking into consideration the patient’s general condition. Basically, during the study period, neoadjuvant chemotherapy or neoadjuvant chemoradiotherapy was administered to patients with any clinical T stage (cT1–4) and any LN involvement, including regional LNs and distant LNs (M1 lym), without distant organ metastasis [12–14]. Neoadjuvant chemoradiotherapy was considered especially for the locally advanced esophageal cancer [15–17].

In all patients, esophageal cancer diagnosis was confirmed with histopathologic evaluation of the tumor samples and staging was performed according to the 8th edition of the UICC TNM classification.

### Surgical procedures

All patients underwent standard surgery including subtotal esophagectomy with two- or three-field LN dissection, which was performed via right thoracotomy or video-assisted thoracic surgery [18] and gastric tube reconstruction, according to the Japanese Classification of Esophageal Cancer [19].

### Follow-up

All patients were followed with outpatient clinic visits at 3–4 month intervals during the first 2 years and every 6 months for years 3–5. CT scans were evaluated every 3–4 months during the first 2 years and every 6 months for years 3–5. Annual upper gastrointestinal endoscopy was performed to screen for recurrence at the anastomotic site and gastric conduit. In cases where CT findings indicated recurrence, further evaluations were performed using more selective methods, such as PET–CT, bone scintigraphy, and magnetic resonance imaging.

### Diagnosis of LN metastasis

In addition to CT, PET–CT was used for the diagnosis and staging of LN metastasis, if necessary. Briefly, LNs with a long diameter of ≥ 8 mm by CT or positive LNs with a maximum standardized uptake value of ≥ 2.5 when CT shows a long diameter of 8 mm or more, or LNs with SUVmax ≥ 2.5 by PET–CT, were considered positive [20–23]. All assessments were performed by at least one radiologist and more than two surgeons specialized in esophageal cancer.

### Statistical analysis

The X-tile software version 3.6.1 (https://medicine.yale.edu/lab/rimm/research/software/) was used to determine the optimal cutoff number of metastatic LNs for cancer-specific survival in patients with LN metastasis [24]. Cancer-specific survival was calculated from the date of surgery to the date of death specifically due to esophageal cancer or to the last known date of follow-up. Kaplan–Meier curves were generated to evaluate survival, and the results were analyzed using the log-rank test. Differences were considered statistically significant with a *p* value of < 0.05. All statistical analyses were performed using JMP version 17.0 (SAS Institute, Cary, NC, USA).

## Results

### Patient characteristics

Among a total of 962 patients with esophageal cancer in the database, 43 patients who underwent noncurative resection, 65 patients with insufficient information to determine cN and pN staging, and 79 patients with incomplete follow-up information due to care in other institutions were excluded. The final study cohort included 775 patients, including 643 male and 132 female patients, who were retrospectively analyzed.

The cohort characteristics are described in Table 1. The mean patient age was 66.4 ± 8.6 years. Middle thoracic esophagus was the most common primary tumor location (44%), 480 patients underwent preoperative therapy, cervical LN dissection was performed in 559 patients, and the histologic type was squamous cell carcinoma in 93% of the cases. According to the 8th edition of the UICC TNM staging, 89 and 72 patients were diagnosed with cM1 or pM1 cancer, respectively, primarily due to supraclavicular LN metastasis.

**Table 1:** Demographic and clinical characteristics of the study cohort

Sex	Male/female	541/114
Age		67 (39–90)
Location	Ut/Mt/Lt	109/284/262
cT	1/2/3/4a/4b	238/117/261/6/33
cN	0/1/2/3	294/253/84/24
cM	0/1	655/0
cStage	0–1/2/3/4	230/116/246/63
pT	0/1/2/3/4	50/306/84/205/10
pN	0/1/2/3	345/193/85/32
pM	0/1	658/0
pStage	0–1/2/3/4	244/178/192/41
Field of LN dissection	2F/3F	206/445
Preoperative therapy	Yes/no	390/265

*LN* lymph node, *2F* two-field lymph node dissection, *3F* three-field lymph node dissection

### Optimal cutoff values for the number of LNs to determine cN and pN stages

**Fig. 1:**
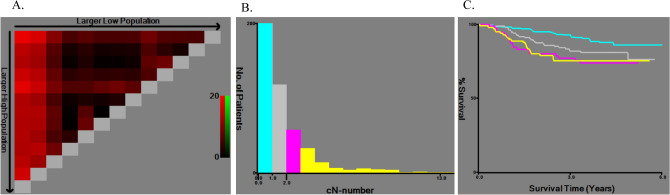
X-tile analysis of cancer-specific survival after esophagectomy for clinical N staging. X-tile plots showing Chi-square estimates with cutoff values for clinically determined metastatic lymph nodes (LNs) to create low, medium, and high clinical node (cN) stages. **a** In patients with LN metastasis, the optimal cutoff values were 2 and 3 for the number of metastatic LNs at the maximum Chi-square value of 16.3. **b** Histogram of the overall study cohort divided into subgroups according to the optimal cutoff value of the metastatic LNs based on X-tile analysis (0, 1, 2, and ≥ 3). **c** Kaplan–Meier curves for cancer-specific survival in groups stratified using the optimal cutoff values for clinical LN metastasis. Blue curve represents patients without LN metastasis, gray curve represents patients with 1 metastatic LN, pink curve represents patients with 2 metastatic LNs, and yellow curve represents patients with ≥ 3 metastatic LNs

**Fig. 2:**
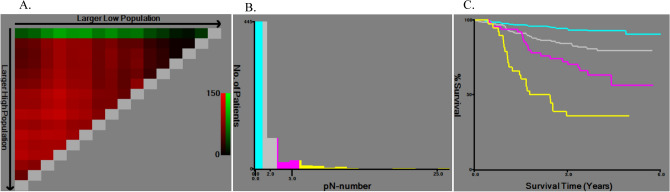
X-tile analysis of cancer-specific survival after esophagectomy for pathologic N staging. X-tile plots showing Chi-square estimates with cutoff values for pathologically determined metastatic LNs to create low, medium, and high pathologic node (pN) stages. **a** In patient with LN metastasis, the optimal cutoff values were 3 and 7 for the number of metastatic LNs at the maximum Chi-square value of 114.5. **b** Histogram of the overall study cohort divided into subgroups according to the optimal cutoff value of the metastatic LNs based on X-tile analysis (0, 1–2, 3–6, and ≥ 7). **c** Kaplan–Meier curves for cancer-specific survival in groups stratified using the optimal cutoff values for pathological LN metastasis. Blue curve represents patients without LN metastasis, gray curve represents patients with 1–2 metastatic LNs, pink curve represents patients with 3–6 metastatic LNs, and yellow curve represents patients with ≥ 7 metastatic LNs

The optimal cutoff values for the number of LNs to diagnose LN metastasis were determined using the X-tile software. Based on the minimal *p* value approach, 1, 2, and 3 metastatic LNs were identified as candidate cutoff values to test their feasibility in cN staging, and the maximum Chi-square log-rank value was 16.3 for cancer-specific survival in cN-positive patients (Fig. 1). Using the minimal *p* value approach, 1, 3, and 7 metastatic LNs were identified as candidate cutoff values to test their feasibility in pN staging, and the maximum Chi-square log-rank value was 114.5 for cancer-specific survival in pN-positive patients (Fig. 2). Of note, the cutoff values for pN staging were identical to those outlined in the 8th edition of the UICC TNM classification.

### Relationship between the number of metastatic LNs and prognosis according to the modified cN and pN staging

**Fig. 3:**
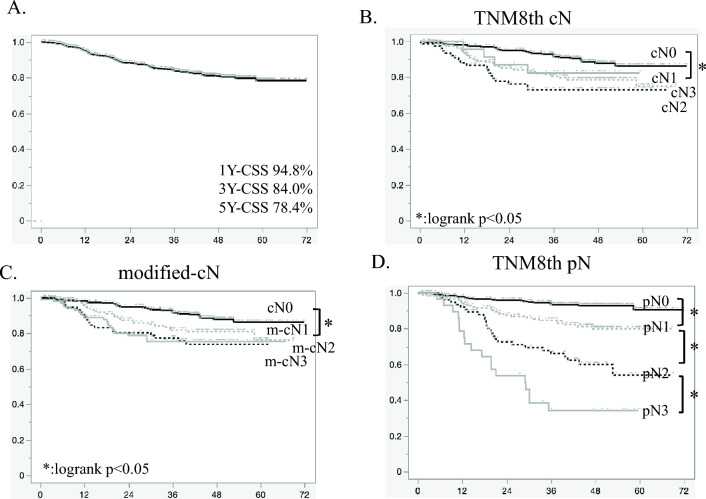
Cancer-specific survival curves. Kaplan–Meier survival curves for **a** all patients and **b**–**d** patients stratified by **b** conventional cN stage, **c** modified cN stage, and **d** conventional pN stage. CSS, cancer-specific survival

In the overall cohort, the 1-, 3- and 5-year cancer-specific survival rates were 94.8%, 84.0%, and 78.4%, respectively (Fig. 3a). First, we evaluated the impact of cN staging on prognosis based on the conventional cutoff values for the number of metastatic LNs (cN0 [0 LN], cN1 [1–2 LNs], cN2 [3–6 LNs], and cN3 [≥ 7 LNs]). Our analyses revealed that the prognosis was significantly better in patients with cN0 disease than in those with cN1 disease (*p* = 0.0017). However, no significant differences in prognosis were found among the patients with cN1, cN2, and cN3 disease (cN1 vs cN2, *p* = 0.2328 and cN2 vs cN3, *p* = 0.3198) (Fig. 3b). By X-tile analysis, the patients were reclassified into the following four stages according to the modified cutoff number of metastatic LNs (m-cN): cN0 (0 LN), m-cN1 (1 LNs), m-cN2 (2LNs), and m-cN3 (≥ 3 LNs). Our analyses indicated that the prognosis was significantly better in patients with cN0 disease than in those with m-cN1 disease (*p* = 0.0211), although no significant differences in prognosis were observed among the patients with m-cN1, m-cN2, and m-cN3 disease (m-cN1 vs m-cN2, *p* = 0.2232 and m-cN2 vs m-cN3, *p* = 0.9193), even when the best cutoff was used (Fig. 3c).

We next examined the impact of the conventional cutoff values used for pN staging, which were identical to those determined in the present study (pN0 [0 LN], pN1 [1–2 LNs], pN2 [3–6 LNs], and pN3 [≥ 7 LNs]), on prognosis. As shown in Fig. 3d, the prognosis was significantly different among the patients with pN0, pN1, pN2, and pN3 disease (pN0 vs pN1,* p* < 0.0001; pN1 vs pN2,* p* < 0.0001; and pN2 vs pN3,* p* < 0.0001).

### Impact of neoadjuvant therapy on prognostic stratification of prognosis according to the modified cN and pN staging

**Fig. 4:**
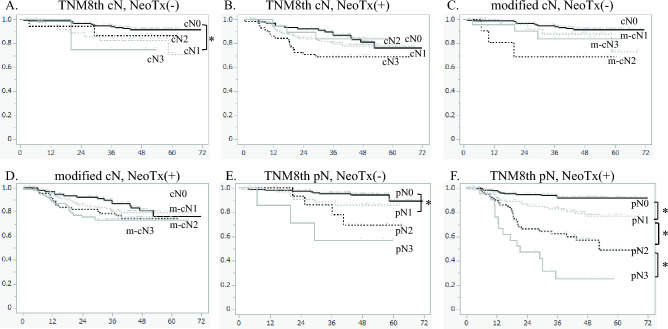
Cancer-specific survival curves according to neoadjuvant therapy. Kaplan–Meier survival curves for **a** all patients and **b**–**d** patients stratified by **b** conventional cN stage, **c** modified cN stage and **d** conventional pN stage. NeoTx, neoadjuvant therapy

Next, we examined the impact of neoadjuvant therapy on prognosis based on the stratification of patients according to the currently used cN staging. In patients who did not receive neoadjuvant therapy, the prognosis was significantly better in patients with cN0 disease than in those with cN1 disease (p = 0.0348). However, the prognosis was not significantly different among the patients with cN1, cN2, and cN3 disease (cN1 vs cN2,* p* = 0.7164 and cN2 vs cN3,* p* = 0.5683) (Fig. 4a). Conversely, in patients who received neoadjuvant therapy, the prognosis was not significantly different among the patients with cN0, cN1, cN2, and cN3 disease (cN0 vs cN1,* p* = 0.3601; cN1 vs cN2,* p* = 0.1029; and cN2 vs cN3,* p* = 0.1856) (Fig. 4b).

Next, we examined the impact of neoadjuvant therapy on prognosis in patients stratified using the modified cN staging. In patients who did not receive neoadjuvant therapy, the prognosis was not significantly different among those with cN0, m-cN1, m-cN2, and m-cN3 disease (cN0 vs m-cN1,* p* = 0.2608; m-cN1 vs m-cN2,* p* = 0.1787; and m-cN2 vs m-cN3;* p* = 0.2905) (Fig. 4c). Similarly, in patients who received neoadjuvant therapy, the prognosis was not significantly different among those with cN0, m-cN1, m-cN2, and m-cN3 disease (cN0 vs m-cN1,* p* = 0.5675; m-cN1 vs m-cN2,* p* = 0.4425; and m-cN2 vs m-cN3,* p* = 0.7111) (Fig. 4d).

Finally, we examined the impact of neoadjuvant therapy on prognosis in patients stratified using the conventional pN staging. In patients who did not receive neoadjuvant therapy, the prognosis was significant different among those with pN0 and pN1 disease (pN0 vs pN1,* p* = 0.0917), but the prognosis was not significantly different among those with pN1, pN2, and pN3 disease (pN1 vs pN2,* p* = 0.3184; and pN2 vs pN3,* p* = 0.3763) (Fig. 4e). Conversely, in patients who received neoadjuvant therapy, the prognosis was significantly different among those with pN0, pN1, pN2, and pN3 disease (pN0 vs pN1,* p* = 0.0025; pN1 vs pN2,* p* = 0.0046; and pN2 vs pN3,* p* = 0.0104) (Fig. 4f).

## Discussion

The main difference between the UICC TNM staging system for esophageal cancer, which is used worldwide, and the Japan Esophageal Society Japanese Classification of Esophageal Cancer (JCEC), which is used primarily in Japan, is the definition of N staging. In the UICC TNM staging system, N staging is based on the number of intraregional LN metastases regardless of the tumor site. Conversely, the 11th edition of the JCEC has adopted a detailed system to determine the N stage based on tumor location and the site of LN metastasis using the efficacy index of LN dissection [19, 25]. In gastric and colorectal cancers, N staging in Japanese classifications has been revised to match that used by the UICC TNM staging system. Several studies have reported that the prognosis of gastric cancer is more closely associated with the number of metastatic regional LNs than with the anatomical position of the metastatic LNs [26, 27]. Before the 7th edition of TNM staging system for esophageal cancer, N staging simply included N0 (no LN metastasis) and N1 (LN metastasis present). Eloubeidi et al. suggested the addition of two other important factors, including tumor length and the number of metastatic LNs, to the TNM staging system for esophageal cancer [28]. N staging underwent the most notable redefinition in the 7th edition of TNM staging system for esophageal cancer and included N0–N3 stages according to the number of metastatic LNs. Talsma et al. reported that overall survival prediction was significantly improved using the pT, pN, and pM stages for stratification based on the 7th edition compared to that based on the 6th edition [29]. Similarly, in patients with esophageal cancer treated with neoadjuvant chemotherapy followed by esophagectomy, survival probability differed among patients in different ypN stages after neoadjuvant treatment based on the 7th edition [30]. Conversely, in a study using the 7th edition of the UICC TNM staging system, Yamasaki et al. reported no significant survival differences among the pN2, pN3, and M1 subgroups of patients with esophageal cancer [31]. Further, Ning et al. found no significant survival difference between the pN2 and pN3 subgroups defined by the 7th edition of TNM staging system. However, when the authors applied a modified staging approach based on the number of metastatic LN fields (pN0, no metastatic LNs; pN1, metastatic LN in 1 field; pN2, metastatic LNs in 2 fields; pN3, metastatic LNs in > 2 fields), the survival difference between the refined pN2 and pN3 groups could be discriminated well, suggesting that both the number and the extent of LN metastasis could provide a better basis for distinguishing subgroups of patients with different prognoses after radical esophagectomy [32]. Ozawa et al. reported that N staging based on the 8th edition of the UICC TNM staging system tended to be a more precise indicator of survival compared to the 11th edition of the JCEC, especially for lower thoracic esophageal tumors [33]. Thus, the prognostic performance of the number of metastatic LNs as delineated in the 7th edition of the UICC TNM staging system varies across studies, with no consensus regarding the appropriate cutoff number of metastatic LNs.

We compared the performance of N staging based on the site and number of metastatic LNs in a large cohort of patients in a multicenter setting. Our analyses suggest that the pretreatment cN stage is sufficient to determine the presence of metastasis and that the modified N staging system, including m-cN1, m-cN2, and m-cN3, may not improve prognostic stratification, given that the modification did not clearly distinguish prognostic groups. Successful discrimination between cN-negative and cN-positive patients is important for the optimal implementation of treatment plans. In the present study, 35% of the patients who were diagnosed with cN0 disease before treatment had pathologic LN metastases as well; thus, advances in imaging technologies are warranted to improve the diagnosis of patients with cN-positive disease. In addition, preoperative treatment is the standard approach and the number of pathologic LN metastases may not always match the anticipated number of LN metastases before treatment. In fact, the Japanese Classification of Gastric Carcinoma subclassifies the cN stage into cN-negative or cN-positive in the classification of progression. In other words, considering the limitations of preoperative diagnosis, N staging is based solely on the presence or absence of LN metastasis. For use related to preoperative chemotherapy and clinical trials, the term cN-positive, i.e., metastasis in regional LN nodes, is accepted in determining stage without counting the number of positive LNs. Conversely, our analyses revealed that the number of pathologic metastases was an important factor in predicting prognosis and the conventional classification might have the best prognostic value. The correlation between the number of cN and the number of pN was lower in patients who received preoperative treatment than in those who did not receive preoperative treatment. This finding suggested that the performance of pN staging in prognostic prediction was superior to that of cN staging due to the influence of preoperative treatment.

In the present study, the methods used to determine LN metastasis were based on the overall judgment of clinicians in each facility and not on uniform criteria, which was a major study limitation. However, our findings are based on real-world data. Second, the results were obtained from a large cohort treated in high-volume centers in recent years. The overall prognosis of the study cohort might be better than previously reported rates because of recent advances in chemotherapy and surgery.

In summary, in the present study investigating the optimal cutoff values for the number of metastatic LNs in staging patients with esophageal cancer, our analyses confirm that the currently used cutoff values remain valid for prognostic stratification despite the incorporation of preoperative chemotherapy into standard care by the large thoracic esophageal cancer cohort.
